# Immunogenicity of Anti-TNF-α Biotherapies: I. Individualized Medicine Based on Immunopharmacological Evidence

**DOI:** 10.3389/fimmu.2015.00152

**Published:** 2015-04-08

**Authors:** Klaus Bendtzen

**Affiliations:** ^1^Institute for Inflammation Research (IIR 7521), Rigshospitalet University Hospital, Copenhagen, Denmark

**Keywords:** anti-TNF-α biopharmaceuticals, immunogenicity, anti-drug antibodies, pharmacokinetics, pharmacodynamics, theranostics, individualized medicine

## Abstract

Specific inhibition of the cytokine, tumor necrosis factor-α (TNF), has revolutionized the treatment of patients with several autoimmune diseases, and genetically engineered anti-TNF antibody constructs now constitute a heavy medicinal expenditure in many countries. Unfortunately, up to 30% of patients do not respond and about 50% of those who do loose response with time. Furthermore, safety may be compromised by immunogenicity with the induction of anti-drug-antibodies (ADA). Assessment of drug pharmacokinetics and ADA is increasingly recognized as a requirement for safe and rational use of protein drugs. The use of therapeutic strategies based on anti-TNF drug levels and ADA rather than dose-escalation has also proven to be cost-effective, as this allows individualized patient-tailored strategies rather than the current universal approach to loss of response. The objective of the present article – and the accompanying article – is to discuss the reasons for recommending assessments of circulating drug and ADA levels in patients treated with anti-TNF biopharmaceuticals and to detail some of the methodological issues that obscure cost-effective and safer therapies.

## Introduction

Treatment of patients with various immunoinflammatory diseases has benefited considerably from the use of genetically engineered antibodies that specifically target inflammatory cytokines and cytokine receptors. The first example was the use of antibody constructs that specifically antagonize the human cytokine, tumor necrosis factor-α (TNF). In most cases, these biopharmaceuticals dramatically improve the conditions of patients with diseases such as rheumatoid arthritis (RA) and ankylosing spondylitis, Crohn’s disease (CD) and ulcerative colitis, psoriasis and suppurative hidradenitis, and diabetic macular edema and age-related macular degeneration of the eye (1).

The currently used anti-TNF-biopharmaceuticals include the chimeric human/mouse mAb, infliximab (Remicade^®^), the human mAbs, adalimumab (Humira^®^) and golimumab (Simponi^®^), the mAb/Fab fragment, certolizumab pegol (Cimzia^®^), and the human TNF receptor/IgG1 fusion protein, etanercept (Enbrel^®^), and corresponding recently marketed biosimilars. They all counteract the effects initiated through TNF binding to cellular TNF-receptors. The effects have been dramatic in a large number of patients, and anti-TNF biopharmaceuticals now constitute one of the heaviest medicinal expenditures in many countries.

## Inadequate Effect of TNF-Antagonists

The use of anti-TNF biopharmaceuticals has faced physicians with a number of challenges. Apart from immunosuppression and other side-effects, up to 50% of patients suffer loss of response (LOR) (1–5). In these cases, physicians are left with few choices, all based on clinical outcome. Most often they choose to intensify treatment with the existing drug, or they switch to another TNF-inhibitor or to a different class of drugs. This has several disadvantages. Patients with disease symptoms in the presence of otherwise therapeutic drug levels are not identified (see below), and symptoms and tissue damage continue if the new and empirically chosen drug is also ineffective.

The mechanisms underlying LOR are usually unknown, partly because the problem has been neglected in the past. Possible explanations include insufficient compliance/bioavailability and pharmacokinetic (PK) as well as pharmacodynamic (PD) issues. One way to deal with these problems is to use therapeutics diagnostics, “theranostics.” This may enable physicians to identify patients for whom a medication or a change in medication is likely to work. It also provides an opportunity to tailor anti-TNF therapies according to individual needs in contrast to the currently recommended generic approach. Testings for anti-drug antibodies (ADA) may also inform of the risk of potentially dangerous reactions caused by drug immunogenicity. Finally, theranostics has the potential to reduce the cost of these expensive therapies (6).

## Rational Therapies Based on Pharmacokinetic Evidence

There is now plenty of evidence that long-term use of biological TNF-inhibitors benefits from knowledge of the fate of the drugs in individual patients and whether or not a drug induces ADA (1). Trough serum levels of drugs are surrogate PK-markers. These levels vary markedly between patients, and they often differ over time even in the same individual (7, 8). These differences depend not only on the structure and formulation of the TNF-antagonists, and route and frequency of administration, but also on patient characteristics, for example, age, sex, weight, underlying and intercurrent diseases, parallel medications, and individual immune responsiveness. If patients with LOR are monitored for circulating drug levels, one would be able to adjust treatment intensity on the basis of pharmaceutical evidence. In patients with low TNF-neutralizing capacity, higher dosage or more frequent administration of the drug would be logical, provided there are no ADA. On the other hand, some patients with primary LOR have therapeutic or in some cases very high drug levels, and these patients are not likely to benefit from intensified therapy (9). Changing to another TNF-antagonist is also likely to be ineffective, because these patients are unresponsive despite already high anti-TNF activity in the blood. Interestingly, the findings of supra-therapeutic concentrations of drug in patients with LOR raise the question if TNF is a pathogenetic factor in all patients with the same diagnosis (10). Nonetheless, monitoring functionally active drugs is warranted in patients with primary LOR because demonstration of high drug levels, and absence of ADA, would allow early change to effective treatment, thus saving months of useless and expensive therapies.

## Safer Therapies Based on Testings for Drug Immunogenicity

Sooner or later, many patients treated with anti-TNF biopharmaceuticals experience side-effects or LOR (11). In some cases, LOR can be related to individual differences in bioavailability and to mechanisms underlying inflammation in the affected tissues, including infections and changes in companion therapies. A major contributor to secondary response failure, however, is drug immunogenicity with production of ADA that neutralizes the drug’s TNF-antagonistic effect and/or clears the drug from the circulation (2). In RA and CD patients, for example, half the patients with initial response to infliximab suffer LOR at later stages, and ADA have repeatedly been shown to associate with LOR development. This frequency may even be a low estimate, because the full impact of drug immunogenicity is realized only if patients are monitored for ADA on a routine basis or every time side-effects or treatment failure occurs. If not, as is the usual situation today, physicians will never know that ADA could be the cause of side-effects and LOR.

**Figure 1:**
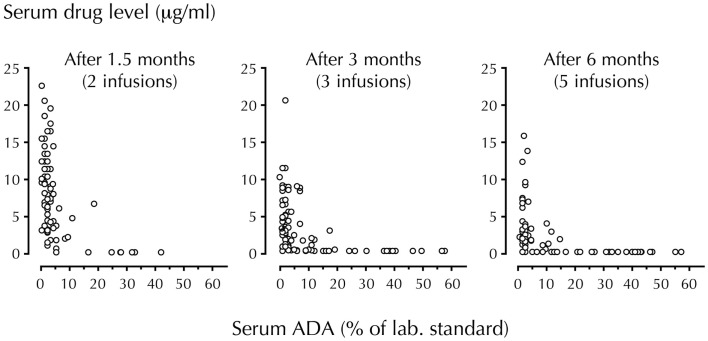
**Temporal association of circulating drug (infliximab) and ADA levels**. Trough serum levels of infliximab and anti-infliximab antibody (ADA) were measured in 106 RA patients using radioimmunoassay. Infliximab was administered at a dosage of 3 mg/kg. Note the step-by-step disappearance of infliximab when ADA develop (44% of patients were ADA-positive after 6 months). With permission from Arthritis and Rheumatism (7).

It is known that trough serum levels of drug decline as soon as ADA appears. In many cases, the drug disappears completely as documented in RA patients treated with infliximab (Figure 1). Investigations of RA and CD patients have also shown that low serum levels of infliximab, adalimumab, and etanercept, and the presence of ADA correlate with the requirement for dose increase and therapeutic failure. Furthermore, serious side-effects that could be mediated by drug–ADA immune complexes have also been reported. These include infusion and allergic reactions, serum sickness, bronchospasm, arthus reactions, and vasculitis, some with fatal outcome (7, 12). In view of such findings, it is notable that the majority of patients still receive TNF-antagonists without attention to safety issues related to induction of ADA.

An important but often neglected aspect of testing-based therapeutic strategies is the necessity for assays to accurately and reliably report serum levels of active drug and ADA. Assessing ADA, for example, is generally impeded by the fact that most anti-TNF drugs are by themselves immunoglobulin constructs, and by the complexity of detecting antibodies against antibodies in various binding assays; see accompanying article.

## Testing-Based Strategies for Therapeutic Guidance

Some clinicians find that there is little need to monitor drug and ADA levels if patients are doing well on standard regimens. Some also question the importance of ADA development because it is not always accompanied by clinical manifestations; this, however, may be related to the fact that it may take months from drug-neutralization till LOR develops. The use of assays that have little therapeutic relevance, and assays known to generate false-negative results contribute as well; see accompanying article.

The wide use of ELISA for detection of ADA in patient serum is perhaps the most problematic example, as this type of assay cannot detect ADA in the presence of drug (13). Consequently, ELISA and other assays that underestimate ADA in the circulation may contribute to the confusion many clinicians feel regarding drug immunogenicity. The problem has also been acknowledged by regulatory authorities, as both the U.S. Food and Drug Administration and the European Medicines Agency require ADA-screening before accepting therapeutic use of new biological drugs, including biosimilars (http://www.fda.gov/downloads/Drugs/.../Guidances/UCM192750.pdf).

Some clinicians find it sufficient to monitor drug levels alone (without ADA), because ADA should reflect itself in subtherapeutic or undetectable drug levels in the circulation. This is not prudent for the following reasons:

- there are other causes of low drug levels than ADA. These include improper handling and storage that may cause drug aggregation, compliance problems especially when anti-TNF drugs are administered by patients themselves, and blood sampling at the end of longer than normal injection cycles, which, if dosage is unaltered, result in lower than normal drug levels. Degradation and/or elimination of drug by other factors than ADA may also play a role.
- If physicians continue to treat a patient with a drug that has induced ADA, possibly even with increased doses due to low drug levels, prolonged therapy becomes both costly and ineffective; it also increases the risk of adverse events.
- ELISA and other binding assays do not reveal the TNF-neutralizing capacity of an anti-TNF antibody construct. Consequently, high drug levels measured by ELISA may erroneously be interpreted as “therapeutic” even in cases where there is an insufficient TNF-neutralizing activity in the circulation. This may result from small immune complexes consisting of drug bound to functionally monovalent ADA, or drug bound to endogenous TNF, a situation calling for intensified rather than unaltered anti-TNF therapy.

Assuming the use of accurate assays that mirror the *in vivo* conditions, how might “theranostics” help physicians optimize efficacy and safety in individual patients?

In case of LOR, one should determine if the symptoms can be attributed to increased *activity* of the primary disease or to non-inflammatory processes, for example, anatomical complications to the original disease. Or the “new” inflammatory activity might be due to other conditions than relapse of the primary disease, for example infections, ischemia, etc. In case of suspected drug failure, it is advisable to assess the serum levels of both drug and ADA to get insight into the pharmacoimmunology at the time of LOR. A previously proposed decision algorithm considers four principal situations (14), see Table 1.

**Table 1:** **Decision algorithm for patients with LOR to anti-TNF biopharmaceuticals**.

Trough drug level	Trough anti-drug antibodies (ADA) level	
	No	Yes
**Sub-therapeutic (full compliance)**	**(1) Problem**	**(3) Problem**
	Insufficient bioavailability and/or increased non-ADA-mediated clearance, see below	Insufficient bioavailability caused by ADA, including pre-existing anti-murine (Fab) IgG against IFX
	**Treatment**	**Treatment**
	Intensify therapy	Shift to another TNF-antagonist
		Note: Test ADA for cross-reactivity if shifting to biosimilar
**Therapeutic or supra-therapeutic**	**(2) Problem**	**(4) Problem**
	*Inflammation*	*Pharmacodynamic*
	Pharmacodynamic issue (symptoms not driven by TNF?)	Non-functional ADA
	*No inflammation*	*Methodological*
	See below	False positive test
	**Treatment**	**Treatment**
	Confirm inflammatory activity	Consider testing for functionally active, i.e., drug-neutralizing ADA
	*Inflammation*	Treat as in scenario 2
	Anti-TNF drugs are ineffective, shift to non-TNF targeting therapy (surgery?)	
	*No inflammation*	
	Treat the underlying cause	

Therapeutically relevant cut-off levels for drug and ADA should be determined by prior clinical validation (10, 14).

Four scenarios are shown:

1. LOR with sub-therapeutic levels of drug and no ADA. This condition is assumed due to inadequate bioavailability and/or PK-issues with elevated drug turn-over. The latter might be caused by increased inflammatory load with extensive and/or elevated expression of TNF in the affected tissues or drug-losing enteropathy in patients with intestinal inflammation. Patients in this group may benefit from intensified therapy with the already administered anti-TNF medication.
2. LOR in the presence of moderate or even high circulating TNF-neutralizing capacity. This is speculated to constitute a PD-issue originating from activation of new immunoinflammatory pathways that bypass TNF as a pathophysiological factor. These patients are not likely to benefit from intensified therapy with the TNF-inhibitor already used or, indeed, a change to another TNF-inhibitor. These patients should be switched to a non-TNF targeting therapeutic principle.
3. LOR with drug-neutralizing ADA and inadequate TNF-inhibition with or without increased drug clearance. As ADA are almost always drug-specific, these patients may benefit from switching to a different anti-TNF drug. Note that this may not be the case with follow-on medicines (biosimilars). In these cases, a serum sample with ADA directed against the original TNF-antagonist should be tested for cross-reactivity against the biosimilar intended for further therapy.
4. LOR with optimal level of drug and detectable ADA is a rare finding. This situation may be seen when binding assays for ADA are false-positive. It has also been seen when a high-sensitivity binding assay such as homogeneous mobility-shift assay (HMSA) reports low-avidity or otherwise functionally inactive ADA. In such cases, sera should be retested using a cell-based assay capable of detecting functionally active drug and drug-neutralizing ADA (10). In case of unchanged findings, LOR in these patients are considered as a PD-issue that should be treated as in scenario 2.

The algorithm shown in Table 1 has been supported by other investigators (3–5, 15) and has been tested in a prospective and randomized investigation of infliximab-treated CD patients with LOR (6). Compared to the recommended escalation of drug dosage, treatment guided by the algorithm reduced the overall treatment costs by 50% without affecting clinical efficacy.

It is prudent to realize that the immunogenicity of biosimilars may not be the same as that of the original drugs. This is because a biosimilar, even with an aminoacid sequence identical to that of the parent drug, may possess subtle differences for example in glycosylation and pharmaceutical formulation that may affect immunogenicity. ADA testings in patients receiving biosimilars are therefore warranted before and after shift of therapy.

## Conclusion and Perspectives

- About one-third of patients with common chronic immunoinflammatory diseases do not respond to anti-TNF biopharmaceuticals, and another one-third sooner or later experience LOR despite ongoing therapy.
- It is well established that immunogenicity impacts therapies with protein drugs, including TNF-inhibitors, and severe safety issues may result from ADA development.
- PK-issues in connection with ADA development correlate with poor outcome of anti-TNF therapies. This is associated with drug levels that are inadequate to neutralize TNF in the circulation and in tissues affected by the underlying disease.
- Determining optimal therapy in patients with LOR is challenging. The recommended strategy of dose escalation and, if ineffective, change to another TNF-antagonist may take months and increase the risk of irreversible tissue damage – and carry a high cost.
- Monitoring circulating levels of drug and ADA provides essential information for safe and cost-effective interventions.
- Ideally, assays should mimic the *in vivo* situation and report functionality of both drugs (drug-induced TNF neutralization) and ADA (antibody-induced drug neutralization).
- Binding assays, for example ELISA and HMSA, do not reveal the functions of drugs and ADA, and the artificial setup of these assays may limit their usefulness in the clinical setting.
- Screening for ADA is now required for marketing of all new biological drugs, including biosimilars (U.S. Food and Drug Administration and European Medicines Agency).

## Conflict of Interest Statement

Financial support was obtained from the Danish Biotechnology Programme. Within the last 3 years, the author has received speaker fees from Pfizer and Biomonitor, and owns stocks in the latter.
